# Is It Stem Cell or Magic Potion? The Case of Autologous Fat Injection

**DOI:** 10.1002/ueg2.70162

**Published:** 2025-12-24

**Authors:** Yasuko Maeda

**Affiliations:** ^1^ Department of General Surgery ‐ Department of Surgery and Colorectal Surgery Queen Elizabeth University Hospital Glasgow UK; ^2^ University of Glasgow Glasgow UK

One of the most challenging perianal fistulae to treat are those related to ileal pouch–anal anastomosis. The underlying inflammation and the variable configuration of the pouch, as well as its anatomy in relation to the sphincter complex, add layers of difficulty. It is therefore welcome to read the article by Alqaisi et al. [1] reporting that injection of autologous fat offers a reasonable success rate of 48%. The group has consistently reported commendable outcomes of this treatment for challenging perianal fistulae, including those in Crohn's disease [2].

The cornerstone of perianal fistula treatment is adequate drainage, often achieved by making incisions on the skin. If the fistula tract involves the anal sphincters, this will need to be facilitated by insertion of a seton to keep the tract open for drainage, as preserving sphincter muscles is critical to avoid long‐term continence issues. When the sepsis is deep seated (e.g., beyond the levator muscle) or the fistula is laminated with multiple tracts, or indeed in a sinus configuration without an external opening, the standard techniques of incision and drainage or placement of a seton without compromising continence become practically impossible. This is where the treatment of injecting liquefied fat becomes attractive, as it could potentially reach the nooks and hooks of fistula tracts and their surrounding tissues that standard surgical tools cannot reach.

The critical question remains as to how it works. The presumed mechanism is the efficacy exerted by mesenchymal stem cells contained within the harvested fat. At the advent of stem cell use for perianal Crohn's disease, the initial hypothesis was heavily based on their ‘regenerative’ properties—that undifferentiated stem cells would develop into different types of specialised cells that line the fistula tract and eventually fill the cavity. The stemness of the cell, however, is not equivalent to an autoprogrammed ability to differentiate into specific cell types. Over nearly 30 years of stem cell use for perianal fistulae, there has been no histological or radiological study indicating that the cells have replaced the fistula tract with like‐to‐like cell types—for example, that the portion of the tract traversing the sphincters is filled with smooth and skeletal muscle, or that the mucosa is covered with epithelial cells.

It is more likely that stem cells exert their efficacy through paracrine effects that modulate inflammation—not suppressing the inflammation necessary for the healing process, yet preventing its collateral damage. This has been demonstrated in other fields, such as in studies of stem cell treatment for post–myocardial infarction inflammation [3]. Most injected cells, whether fat or stem cells, are known to have limited longevity, with alteration of their immunoproperties over time; it is therefore more plausible that the injected cells activate local host cells to continue the healing process while exerting their own immunofunctional effects [4, 5]. Anecdotally, fat grafts have been used for many years as tissue fillers, particularly after surgery, and have been shown to soften scarring. This also supports the hypothesis of modulation of the inflammatory process.

What is the proportion of stem cells in liquefied fat, and how many cells are actually viable and exerting their efficacy? Do cells other than stem cells—such as neutrophils, macrophages, or soluble factors such as cytokines harvested at the same time—also play a role? We must also note that in the current study 40% of patients were on biologics, and their relevance to the clinical outcome is not clear. The challenge also seems to remain for pouch–vaginal fistulae, as their healing rate was lower compared with fistulae connected to the skin, which may also have better blood supply to aid healing.

Nevertheless, this is an exciting development in a field where many promising treatments have come and gone into oblivion. The only way forward is continued rigorous auditing of outcomes, explicit reporting of the learning curve, and further studies on molecular aspects to elucidate the mechanism.

## Conflicts of Interest

The author declares no conflicts of interest.

## Data Availability

The author has nothing to report.
